# How Do Different Image Modules Impact the Accuracy of Working Length Measurements in Digital Periapical Radiography? An In Vitro Study

**DOI:** 10.3390/diagnostics15030305

**Published:** 2025-01-28

**Authors:** Vahide Hazal Abat, Rabia Figen Kaptan

**Affiliations:** 1 Faculty of Dentistry, Uskudar University, Istanbul 34768, Turkey; 2 Faculty of Dentistry, Yeditepe University, Istanbul 34728, Turkey; figen.kaptan@yeditepe.edu.tr

**Keywords:** digital radiography, digital image processing, endodontics, image enhancement, working length

## Abstract

**Background/Objectives**: This study aims to evaluate the accuracy of digital dental radiography in determining working length in root canal treatment via various image modules. **Methods**: A total of 40 intact single-rooted, single-canal human premolar teeth were examined. Following meticulous cleaning, the teeth were placed in a maxillary premolar socket within a dry human skull. X-ray images were systematically captured via a Carestream RVG digital sensor under standardized conditions. The digital images are processed under five distinct image modules: 1. original module, 2. autoenhancement module, 3. autoenhancement+negative module, 4. negative module, 5. colored module. Three calibrated observers determined the working length of each digital radiograph after the specified image modules were applied. The agreement between the actual working length and the lengths determined by the observers was evaluated via Pearson correlation analysis. A significance level of 0.05 was set for the statistical tests. **Results**: A high level of intra- and interobserver agreement, with a strong correlation between the actual measurements and all image module groups, was obtained (*p* < 0.001). The original image module group demonstrated the highest compatibility (ICC = 0.940, r = 0.912), whereas the colored image module group exhibited the lowest compatibility (ICC = 0.924, r = 0.879) with the actual measurement. **Conclusions**: This study demonstrates the accuracy of digital radiography in determining working length through the application of various image modules, with the original image module exhibiting the closest alignment to actual working lengths. These findings support the continued use and further development of computer-based image processing tools to optimize clinical outcomes in root canal therapy.

## 1. Introduction

In endodontics, the working length (WL) refers to the measurement between a coronal reference point and the point at which canal preparation and obturation procedures should be completed. WL determination is an essential component of root canal treatment [1]. The literature provides clear evidence that WL is a crucial factor in the success of root canal treatment [2,3]. Studies have shown that there is a 12% decrease in success for each uninstrumented millimeter, whereas overextended root canal fillings result in a 62% loss in success [2]. Additionally, teeth with short root fillings have been found to have a 3.1-fold greater likelihood of being correlated with periapical lesions than teeth with proper root filling lengths [3].

Many techniques can be employed to determine the WL. These include the use of radiography, digital tactile sense, the patient’s response to the placement of a file or paper point into the root canal, electronic root canal measurement, and cone-beam computed tomography imaging. In accordance with the recommendations of the European Society of Endodontology, the utilization of an electronic apex locator in conjunction with undistorted periapical radiography is advised for the confirmation of canal length during root canal treatment [4]. Even if some researchers claim that the new generation of apex locators could determine the WL better than radiography, it certainly depends on the accuracy of the radiography method used [5].

The advent of digital radiographic imaging systems has contributed to the growing acceptance of this technology by dental professionals, as they facilitate the production of high-quality and detailed images during endodontic procedures [6,7]. The advantages of digital radiography over conventional radiography include a reduction in radiation exposure, the elimination of chemicals used in film processing, and improvements in archiving and documentation. One significant advantage of digital radiography is its capacity for image manipulation and enhancement, which is performed with the objective of facilitating more accurate diagnoses [6,8].

In the 1980s, digital radiography was introduced in endodontics, leading to the creation of many commercial image enhancement software tools [9]. Image enhancement tools in the context of endodontics are designed to improve the quality and usefulness of digital radiographs to aid in diagnostic and therapeutic planning [10]. The utilization of enhancement tools is a common practice among endodontists for the purpose of interpreting digital periapical radiographs of teeth [11]. The results produced by the enhancement tools are more visually appealing [12]. Nevertheless, there is no scientific evidence to suggest that they can increase the accuracy of WL determination [13]. Additionally, there is currently no consensus in the literature regarding the optimal conditions for determining the WL in digital periapical radiography.

In light of the extensive implementation of digital radiographic systems in endodontics and the continual advancements in these technologies, clinicians need to select the best imaging parameters when deciding on the WL during root canal treatment. This task becomes particularly challenging when anatomical superimpositions, such as those caused by the maxillary sinus and zygomatic bone, impede the visibility of the apex on radiographs. However, a gap in the literature was identified regarding the effect of such superpositions on the measurement of WL on radiographs.

In contrast to the previous studies, which predominantly utilized dry mandibles [14,15,16] or mandible models [17,18] and evaluated WL in the absence of superimpositions, this research sought to assess WL measurements by incorporating the impact of anatomical superimpositions, such as those caused by the maxillary sinus and zygomatic bones, using the maxilla of a dry human skull. The study has been developed to systematically compare five distinct image processing modules in order to identify the optimal image module for accurately determining WL under these challenging conditions, with the dual aim of addressing the specified gap in the literature and helping clinicians quickly and accurately measure WL on digital radiographs in clinical practice.

## 2. Materials and Methods

The study was conducted in accordance with the Declaration of Helsinki, and the protocol was approved by the Ethics Committee of Yeditepe University (No: 2023/1760) on 12 July 2023. Consent for the use of extracted teeth in the present study was obtained from patients whose teeth had been extracted for periodontal reasons. The power analysis was conducted prior to the initiation of the study. The required sample size was calculated, with an alpha-type error of 0.05 at a beta power of 0.80. The sample size was determined using G*Power v3.1 software (University of California, Los Angeles Advanced Research Computing, Los Angeles, CA, USA). The total number of samples indicated as the ideal size required for determining significant differences was 40.

Prior to the study, 40 permanent, single-rooted, extracted human premolar teeth were collected. The teeth that had been extracted due to periodontal or orthodontic considerations underwent meticulous cleaning procedures. Disinfection and cleaning were performed with 2% glutaraldehyde for 2 h, and the teeth were kept hydrated in water, as previously described [19,20]. A superficial root scaling procedure was used to remove residual tissue and establish a uniform root surface. The exclusion criteria consisted of teeth with root canal treatment, calcification, incomplete root formation, resorptive lesions, or fractures. The teeth were analyzed under an operation microscope (Carl Zeiss AG, Jena, Germany), and teeth with any fractures/cracks, internal/external root resorption, calcification, root canal filling, incomplete root formation, or severe curvature (exceeding 15° based on the Schneider method [21]) were excluded from this study. Teeth whose root lengths were outside the range of 14–17 mm were excluded from the study.

In the preparation of anatomic access cavities, round burs (#010) (Dentsply Maillefer, Ballaigues, Switzerland) were used with a high-speed handpiece. The Gates-Glidden drills (#3) (Dentsply Maillefer, Ballaigues, Switzerland) were employed for the purpose of expanding the coronal and middle portions. The actual WL of the root canal was measured by inserting a size #10 K-file into the canal until the file tip became visible at the apical foramen, as seen under 4 times magnification using an operating microscope (Axiocam 503 color, Zeiss, Jena, Germany). At this point, the silicone stop was placed at the reference point, and the file was then removed from the canal. The distance from the base of the silicone stop to the file tip was measured twice with a digital caliper with 0.01 mm precision. The mean of the two values was then taken as the actual WL.

**Figure 1:**
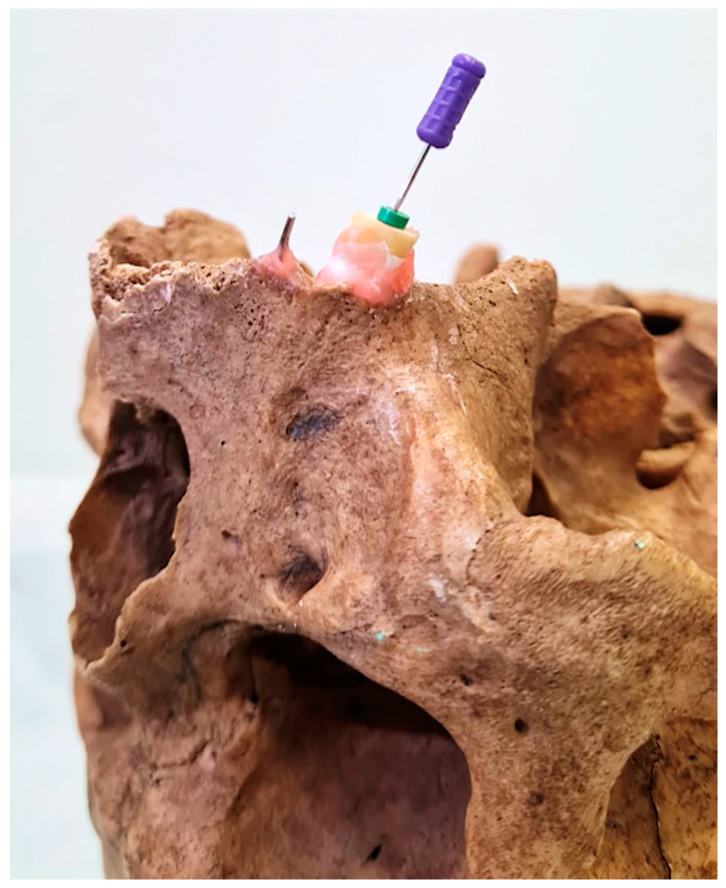
A fixed sample tooth in the maxillary socket along with an adjacent wire.

**Figure 2:**
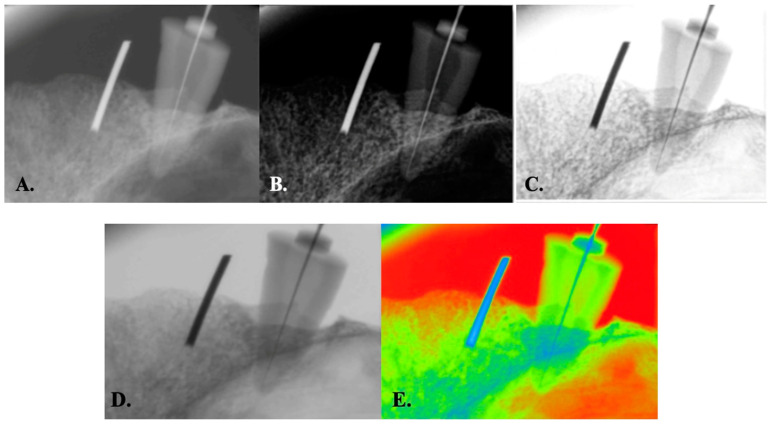
The radiographic images were obtained via five image modules: original (**A**), autoenhancement (**B**), autoenhancement+negative (**C**), original+negative (**D**), and colored (**E**) modules.

The files were subsequently reintroduced into the root canals at the recently determined length and firmly affixed via wax. The roots of the sample teeth were encased with Teflon tape, with three turns applied to represent the periodontal space on the X-ray accurately. Afterward, to mimic clinical scenarios, the samples were placed in the appropriate socket on the maxilla of a dry human skull. A circular orthodontic wire 10 mm in diameter was inserted into the neighboring dental socket and fixed using wax to eliminate radiography magnification (Figure 1). To ensure that all samples were captured under consistent geometric conditions and achieve parallelism during radiographic imaging, the digital RVG 5200 sensor (Carestream Dental, Atlanta, GA, USA) was securely affixed to the palatal side of the sockets in the maxilla. Owing to the palatal curvature of the dry maxilla we used and the necessity to achieve superimposition of the maxillary sinus and zygomatic bone with the root of the upper premolar, a film holder could not be placed; however, to serve the same purpose, the sensor was covered with wax from behind and fixed palatally in the determined position. The source-to-object distance was standardized as 30 cm. An intraoral X-ray unit (New Life Radiology Timer X3000-2C, S.R.L., Grugliasco, Italy) operating at 60 kV, 7 mA, and 0.03 s was used. Following the X-ray procedure for each tooth sample, the KODAK Dental Imaging Software version 6.12.32.0 program (Eastman. Kodak Co., Rochester, NY, USA) was configured to operate in five distinct image modules (Figure 2): A. original module (O), B. autoenhancement module (E), C. autoenhancement+negative module (E–N), D. negative module (N), E. colored module (C). As a result, 200 images were acquired for evaluation by applying five different image modules to 40 teeth. These image modules refer to the system’s built-in components within the software, which, when applied, automatically convert the image to the respective module. The resulting versions of the images were subsequently stored as TIFF files in 5 different image modules.

Three endodontists, with an average age of 40 years (ranging from 33 to 58) and an average clinical experience of 18 years (ranging from 10 to 36), underwent calibration. This calibration was conducted with the known reference length (10 mm) of the 40 orthodontic wires. To provide intraobserver calibration, a calibration test was conducted for each of the observers on two separate occasions, with a time interval of 10 days. Following the completion of both intra- and interobserver calibrations, the observers marked the most apical tip of the file and the corresponding apical tip of the rubber stopper on each radiographic image. Additionally, they marked the most coronal and apical points of the orthodontic wire placed in the adjacent dental socket. In order to ensure the standardization of the observations and to eliminate potential biases in the measurements, the observers were not permitted to adjust the brightness, contrast, or other parameters during the evaluation process.

The observations were conducted in a darkened room to minimize glare, and a consistent computer monitor (HP ProDisplay P17A 17-inch LED monitor, 1280 × 1024 pixels, 60 Hz) (Hewlett-Packard Development Company, L.P, New York, NY, USA) and computer light were employed for standardization.

The fourth observer measured the distance between the points marked by the observers for both the endodontic file and the orthodontic wire. For this measurement, the fourth observer used the distance measurement tool in the Kodak dental imaging software program version 6.12.32.0. The magnification coefficient for each image was calculated by dividing the length of each orthodontic wire measured in the digital radiograph by its actual length (10 mm). To eliminate the effect of magnification, the length of each endodontic file on the radiographic images was divided by the magnification coefficient. All the data were documented on a spreadsheet via Microsoft Excel 2007.

### Statistical Analysis

The normality of the data distribution in all groups was confirmed by the Shapiro–Wilk test. To assess the compatibility of measurements both within and with actual values, we utilized interclass and Pearson correlation tests, alongside observational scatter plot graphics. Data analyses were performed via IBM SPSS Statistics 25 software, with a significance level (α) of 0.05 applied in all analyses.

## 3. Results

**Figure 3:**
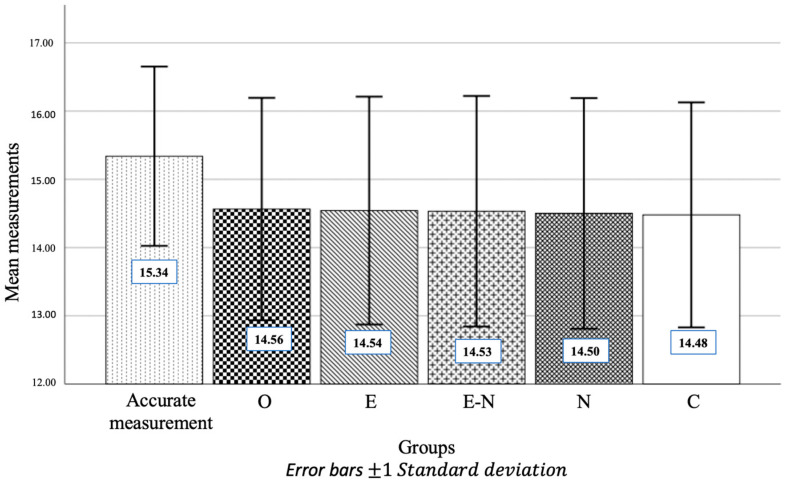
A bar graph showing the mean measurement values for each group.

In this study, X-ray measurements were conducted by three observers via five different image modules. Interobserver agreement was assessed via the interclass correlation, which revealed a very high level of agreement. Additionally, the intraclass correlation coefficient showed excellent intraobserver agreement for all image modules, with means higher than 0.98. The descriptive statistics are presented in Table 1. An illustration of the average values of the measurements for each group is shown in Figure 3.

When the compatibility between actual measurements and those obtained from five different image module groups was assessed, a remarkably strong and statistically significant correlation was observed across all groups (*p* < 0.001). This finding indicates that the actual measurements and all image module group measurements are very compatible with one another. In accordance with the data obtained from the correlation analyses, the original image module group demonstrated the highest level of correspondence (ICC = 0.940, r = 0.912), and the colored image module group demonstrated the lowest level of correspondence (ICC = 0.924, r = 0.879) with the actual measurements (Table 2).

**Figure 4:**
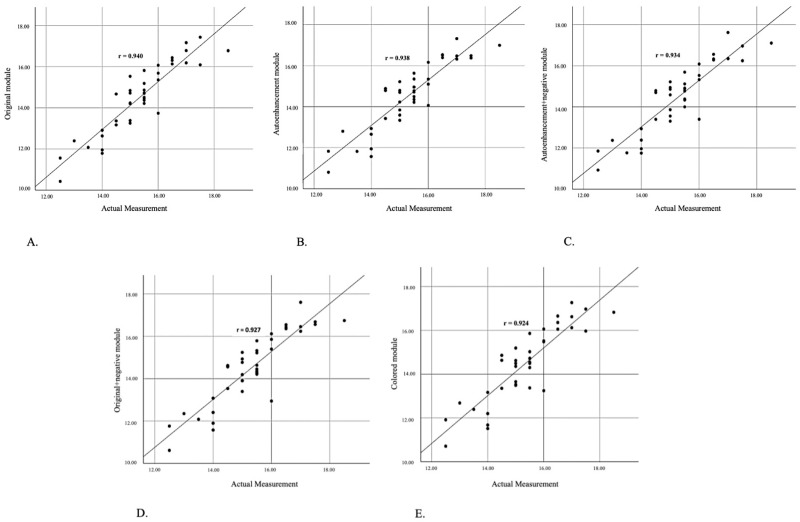
The scatter plot graph illustrates the linear relationship between actual measurements and measurements in the original (**A**), autoenhancement (**B**), autoenhancement+negative (**C**), original+negative (**D**), and colored (**E**) modules.

In fact, when the scatter plot graphics (Figure 4) that show the linear relationship between the five image module groups and the actual measurements are examined, it is possible to see that the points for the measurements between 14 and 16 mm, particularly for the original group, are distributed closer to the correlation line (Figure 4A). This is evident when the scatter plot graphics that show the linear relationship between the various image module groups and the actual measurements are examined.

## 4. Discussion

In endodontic procedures, accurate determination of the WL is pivotal for achieving optimal healing and successful root canal treatment. An inadequate WL may result in insufficient cleaning of the root canal, whereas an excessive WL may lead to overinstrumentation, irritating the periapical tissues. Consequently, inaccurate determination of the WL frequently results in failure of root canal treatment and may necessitate extraction of the tooth, thereby resulting in irreversible consequences for the patient [22].

A variety of techniques have been employed for the determination of WL, including those based on tactile sense, radiographic examinations, the paper point technique, electronic apex locators (EALs), and combinations of these methods. Although several methodologies exist, periapical radiography remains one of the most preferred techniques in contemporary practice [23]. The present study focuses on the evaluation of the impact of different image modules on WL determination through the utilization of radiographic methods. While EALs are more precise in certain clinical scenarios with a reported accuracy of up to 95% [24], radiographs continue to play an essential complementary role [25]. This is particularly the case when EALs have limitations in specific cases, such as extremely wide or obliterated canals [26], or in cases of apical anatomical complexities, where EALs can yield inappropriate readings [27]. Furthermore, radiographic imaging allows clinicians to confirm the integrity of the periapical area, determine the proximity of anatomical structures, and detect potential complications, such as broken files in root canals or root fractures, which are beyond the scope of EALs.

Numerous studies have indicated that the precision of radiographic methods for determining WL relies on the radiographic technique employed [28,29,30,31,32]. Sheaffer et al. [32] reported that higher-density radiographs were necessary for determining the WL. While radiographic imaging constitutes a crucial and indispensable aspect of root canal treatment, there is an ongoing imperative to minimize exposure to ionizing radiation whenever feasible [33]. The adoption of alternative adjunctive methods that maximize benefits while minimizing risks is advantageous [33]. In the context of contemporary practice, the various image processing tools available in digital imaging radiography allow for a clearer visualization of root canal details, providing a more detailed image without the need to increase radiation exposure. The results of a survey study conducted by Madaraati [34] that included 550 general dentists revealed that the contrast tool, an image processing tool, was used in 84% of all patients.

Despite the numerous image processing tools available in X-ray imaging programs, a specific standard has yet to be established for configuring parameters to ascertain the WL. It is highly important to conduct studies that assess the performance of digital radiography under various image modules and evaluate observers’ preferences regarding image quality in the determination of WL. This is because such studies contribute to the establishment of a standardized approach in which the image module most accurately represents the WL. In light of the aforementioned considerations, the present study aimed to evaluate the accuracy of WL measurements by utilizing a range of image processing modules within digital radiography.

As the thickness of the file increased and encompassed a greater number of pixels within the digital radiographic image, the detectability of the file tip improved, thereby facilitating more precise length measurements. In a clinical setting, the determination of WL may prove challenging owing to the difficulty in discerning small file tips. The type of image receptor, the influence of scattered radiation, superimposition of trabecular bone patterns, bone processes, superpositions of roots, variations in bone density, and optimal exposure time may impact the discernibility of the file tip in radiographic images [35]. In one study, distortions were present in detail, especially at the tips of endodontic files, with a special emphasis on files #06 and #08 [36]. It has been proposed that endodontic files smaller than #10 may be inadequate for accurately determining the WL due to their lack of clarity, which makes it challenging to visualize them on radiographs [37]. Kal et al. [38] demonstrated that the mean error in determining the WL decreases as the size of the file increases. Nevertheless, the use of instrumentation to increase the diameter of the canal to a minimum size of 15 before establishing the WL may result in canal transportation and deviation. In the present study, we addressed this challenge by utilizing an ISO #10 K-file, a size that is commonly preferred by dentists because of its efficacy in treating teeth with especially narrow canals.

Under clinical conditions, the measurement of the WL via periapical radiography poses a challenge because of the superposition of anatomical bone areas, anatomical gaps, sinuses, and multirooted teeth [39]. To address this challenge, one potential approach involves taking radiographic images from many perspectives, utilizing distinct instruments placed within different canals for multirooted teeth [40], or employing various image processing modules integrated with digital imaging software [41]. Upon reviewing the literature, it was observed that all the evaluations concerning the measurement of WL were conducted either on acrylic blocks or on the lower mandibular bone. To evaluate the impact of the superposition of both the zygomatic bone and the maxillary sinus on WL determination via digital radiography, our investigation involved the placement of sample teeth within the upper premolar socket of the maxillary bone, which, to the best of our knowledge, is the first study in the literature.

The study findings indicate that the accuracy of the measurements from all the processed digital images was comparable to that of the actual measurements, in accordance with the results of studies by Kal et al. [38], Melius et al. [42], and Schmitd et al. [43]. Conversely, the findings of the study by Farhadi et al. [15] demonstrated that enhancement estimates the file length to be longer than the original image. The discrepancies between the present study and the aforementioned research may be attributed to variations in the method of tooth mounting. In contrast to the methodology employed by the aforementioned researchers, our approach entailed implanting the sample teeth into the maxilla, as opposed to the mandible. Furthermore, the researchers employed the Scanora software program, version 5.1 (Soredex Corporation, Helsinki, Finland), whereas our study utilized Kodak dental imaging software version 6.12.32.0.

The pixel size, which signifies the level of detail in an image, has an inverse relationship with resolution [44]. In Oliveira et al.’s study [18], a comparative analysis was conducted, observing significantly varied outcomes in relation to distinct digital radiographic imaging systems featuring different pixel sizes for the determination of endodontic file lengths. In a study conducted by Kal et al. [38], a comparison was made between various image algorithms obtained through Digora Optime digital imaging systems. The findings indicated that the threshold-enhanced module significantly yielded the least accurate file measurements, whereas contrast/brightness-adjusted images demonstrated the highest accuracy. The discrepancies between our study and theirs may be attributed to the utilization of different digital imaging sensors. The pixel size specified for the Digora images was 71 μm ± 5% according to the manufacturer’s information [45]. However, in the current study, a Carestream sensor with a pixel size of 19 μm was employed, which led to the conclusion that none of the five various image modules had a discernible impact on the outcome of WL determination.

Notwithstanding the increasing availability of enhancement tools in software applications, it is imperative to evaluate their efficacy objectively. The present study demonstrated that the WLs measured under each image module were consistent with the actual WLs. The findings suggest that, although the original image module may provide slightly more precise results, the other modules are still sufficiently reliable and effective for clinical use. It is vital to emphasize that the absence of statistically significant differences in agreement levels among the modules does not imply that using different modules is unnecessary in clinical settings. Instead, the finding highlights that all of the tested modules offer reliable and reproducible measurements, making them viable options for clinicians. It is crucial to recognize the individual visual perception of observers, which inevitably leads to personal preferences for different image modules. From a clinical perspective, the ability of these image modules to provide accurate and repeatable results without the need for additional X-ray exposure is of significant value, ensuring their practical applicability in different clinical scenarios.

Currently, artificial intelligence (AI) is utilized primarily for the enhancement of dental radiology image analysis, particularly in virtual applications. This encompasses tasks such as the identification of periapical lesions, crown and root fractures, and the determination of the WL [46]. AI programs are trained to analyze digital radiographic images with varying degrees of brightness and radiopacity [47]. The establishment of standardized image processing for the consistent determination of accurate WLs via digital radiography will contribute to the development and utilization of AI methods in endodontics. In this context, the potential for AI to resolve a challenge is promising, as it can accommodate the variation that arises from differences in observer interpretation. Through these and similar studies, it is feasible to identify the most efficient image module that accurately portrays the WL. The introduction of a novel parameter, namely, the image module for ‘WL determination’, to AI programs would subsequently enable AI-assisted digital radiographs to provide clinicians with an almost exact measurement of the WL.

In clinical scenarios, the visual characteristics of the radiographic image may be affected by the soft tissue overlaying the bone [48]. It could be argued that this represents a limitation of the present study in that the ex vivo model prepared cannot fully represent the clinical conditions, despite the use of a dry human maxilla. In consideration of the anatomical curvature of the palate of the dry maxilla that was utilized and the necessity to achieve superimposition of both the maxillary sinus and the zygomatic bone with the root of the upper premolar, it was not feasible to employ a film holder. To address this challenge, the wax was used to stabilize the sensor, thereby enabling precise positioning. This approach may be considered another limitation of the present study.

To fully explore the capabilities of current digital radiography systems, further advanced clinical research is essential. This approach would be beneficial for further research, particularly for investigating filters and software tools designed to enhance detail visualization, especially in cases involving damaged teeth, including external root resorption, periapical lesions, previously treated teeth, and those with severely curved canals.

## 5. Conclusions

Despite the common practice of clinicians adjusting contrast, brightness, and color settings while measuring canal length in digital radiography, our study highlights that the different image modules in the Kodak dental imaging software (version 6.12.32.0) did not significantly affect the accuracy of WL measurement. This finding underscores the reliability of the software in maintaining measurement precision, even with various adjustments, and suggests that practitioners can rely on these tools for accurate assessments in clinical practice.

## Figures and Tables

**Table 1:** Descriptive statistics.

Groups	*N*	Mean	St. Deviation
Actual WL	40	15.34	±1.33
Original	40	14.56	±1.63
Autoenhancement	40	14.54	±1.67
Autoenhancement–Negative	40	14.50	±1.69
Original–Negative	40	14.53	±1.69
Colored	40	14.48	±1.65

**Table 2:** Correlation analysis between actual measurements and the five image module groups.

Image Module Groups	Interclass Correlation		Pearson Correlation	
	ICC	Sig	r	Sig
Original	0.940	0.000 *	0.912	0.000 *
Autoenhancement	0.938	0.000 *	0.903	0.000 *
Autoenhancement–Negative	0.934	0.000 *	0.899	0.000 *
Original–Negative	0.927	0.000 *	0.888	0.000 *
Colored	0.924	0.000 *	0.879	0.000 *

Interclass and Pearson correlation analysis. * *p* value significant at the 0.05 level.

## Data Availability

The datasets used or analyzed during the current study are available from the corresponding author on reasonable request.
